# Fragmentary Clippings

**Published:** 1876-07

**Authors:** S. P. Cutler

**Affiliations:** Memphis, Tenn.


### FRAGMENTARY CLIPPINGS.

BY S. P. CUTLER, M. D., D. D. S., MEMPHIS, TENN.

Boston Chemical Journal, May, 1874, page 71.—Is an article entitled, "Why the Teeth Decay." By Lebor & Rottenstein, translated by Prof. T. C. Chandler, of the Harvard Dental School. Speaking of the causes of decay, they regard them as local and chemical mainly, says: "But acids alone will not account for all the phenomena of caries in the teeth. They play a primary and principal part, making the teeth porous and soft, in this state the tissues having lost their normal consistency, fungi penetrate both the canaliculi of the enamel and of the dentine, and by their growth produce softening and destructive effects, much more rapid than the action of acids alone can accomplish." Says, "Bowditch examined forty different persons of different professions, and living different kinds of life; found in almost all of them vegetable and animal parasites. The parasites were abundant in proportion to the neglect of cleanliness." The writer says, "but the investigations of Lebor & Rottenstein appear to have settled the question beyond a doubt." Although these gentlemen seem to have settled the hash on the subject, they, I believe, were not the first, by any means, to call attention to the chemical and parasitical causes of decay. They first informed us that the enamel and dentine contained canaliculi; this is certainly new to me, though this statement may have been an oversight or misprint or mistranslation.

As to fungi entering the tube after the lime has been dissolved out of dentine by acid action, may be regarded as doubtful, owing to the smallness of these tubes they are as attenuated as the smallest fungi, though different statements have been made. Under the microscope they can not be seen in the tubes beyond the point of decay, rarely in the tubuli of softened dentine. I have examined some hundreds of specimens under the instrument and speak advisedly on the subject.

American Journal of Dental Sciences. Feb. 1875. Article Atrophy of the Teeth. Read before the Virginia State

Dental Association. The writer said there were twenty-nine teeth which he extracted at one sitting, without anything but a stimulant. He says: "The teeth are no where affected with disease, except several carious spots not of a serious character, the teeth are nowhere affected, or loss of structure, save on their cutting edges or grinding surfaces, extending over their sides, uniformly about one line or more around each tooth." Says, "the roots being long, well shaped, white and well developed."

This case was a laboring man's daughter, twenty-one years old. Writer speaks of the enamel as being dark brown, and unsightly, abrupt shoulders, and the enamel pealing off from the dentine in places. This case, then, turned wholly on the score of looks. Why was it necessary to remove the back teeth at all, or even any of the lower teeth? which are generally not much seen; and if it was necessary to replace the upper front teeth, would it not have been better to have pivoted them? Instead of removal would it not have been possible to have improved their appearance by filing off some of the enamel, or removing with emery slabs or the engine? Very similar cases have come under my observation, and the idea of extraction simply for defective enamel never occurred to me as being the proper plan to pursue. No doubt these teeth could have been greatly improved in appearance. Again, would a young man marry this young lady any sooner with her new false teeth in her mouth, than with her natural ones, knowing all the facts in the case? It would seem to me not. Now the day she lost her teeth, I would say that she was made ten years older; though with her pretty new teeth she may look younger.

I regard the loss of all the natural teeth at any age, from whatever cause, as a very serious matter, more especially in a young person. I would hesitate long before removing a single tooth when useful or comfortable, simply on the score of looks, even a front tooth. As a profession, at the present time, we are too reckless of other people's teeth, and slay them broad cast. Nothing personal is intended in the above remarks in any respect.

Dental Register, Jan., 1876. Filling Teeth with Tin. Page 10, Prof. W. says: "When two metals are so situated in the mouth, that the mucus membrane forms a connecting conductor, and the fluids are capable of acting on one of them, galvanic action is established sufficient to decompose any of the binary compounds contained in these fluids. The liberated nitrogen, hydrogen and oxygen form ammonia, and then nitric acid; you also have in the mouth, air, moisture and the decomposing food to assist in its production." White decay, the most formidable variety known, is produced by nitric acid, and here you find all components of the teeth are acted on, disentegrated, as far as the disease extends."

In relation to the first statement, for argument's sake, we will admit to be true; but it certainly must be a very small galvanic battery that an ordinary plug in a tooth would form, in fact, so insignificant that it would not amount to anything practically, and could not decompose any binary, or any other compound, to which any practical chemist would bear witness, and it must necessitate a great stretch of the imagination to arrive at such a conclusion.

Admitting, for argument's sake again, that ammonia and nitric acid are both formed in the mouth simultaneously, the result of this double binary process must necessarily be nitrate of ammonia formed instantaneously, and no free acid left; this salt of ammonia is a neutral salt and could have no action on the teeth whatever. So this imaginary thunder mill in the mouth is entirely harmless, should such a state of things exist in the mouth. This fact must have been overlooked by the Prof., as he must admit the validity of the last statement. If such an acid can be, or is formed in the way suggested, it must be the same in all mouths, and should always cause the white variety of decay, where such fillings as spoken of are present.

We often find different forms of decay in the same mouth; black, brown and white; sometimes a white decay on one side, and a black decay on the other side of the same tooth, at the same time. How can this fact be reconciled with the above theory? But the other day I saw a case of the above description; others are constantly witnessing the same thing. We often see good sets of teeth ruined more or less, by the injudicious use of elixir vitriol and tincture of iron, muriated, corroding the enamel and dentine; this corrosion is in the form of white decay generally, there may be some exceptions.

Dental Cosmos, Feb., 1876, page 88. Subject, Operative Dentistry. This is a good paper in general. I propose noticing one point. The writer says: "When an operator has not the ability to perform, or the patient can not afford first class operations, then it may appear necessary to insert tin foil, cement plombe, a preparation of gutta percha, or, as a last resort, and in preference to extraction, amalgam; but outside of the question of galvanic action and the shrinkage or non-shrinkage of amalgam, it is, after it has been inserted for a time, repulsive to the sight."

The writer puts amalgam as the last resort, if nothing else can be used, this stuff can come in, in preference to extraction. It is to be inferred from the above that in some cases the writer does use amalgam, but gives no rules when to use, or how to use it. From its color line this stuff has to take a back seat and keep out of sight.

Now what chance has a material to recommend itself to favor, when used only in the most extremely doubtful cases, with no particular care or pains taken in its use. Had amalgam received the one-hundredth part the discussion and clinical instruction that gold has, much good might have resulted therefrom.

Pennsylvania Journal of Dental Science. April 1876. Remarks on One of the Causes of Death During the Extraction of Teeth under Chloroform. By T. Leander Brunton, M. D., F. R. S., of St. Bartholomews Hospital, London.

In this able paper, we find Dr. Syms' and others' reasons for giving chloroform freely for surgical and dental operations, and give good reasons, no doubt, why danger often occurs under partial chloroform anæsthesia in the extraction of teeth, when if there had been full doses given no reflex action would have occurred, causing arrest of the heart's action. The fifth nerve, more especially, being peculiarly susceptible to this kind of danger, from its seat in the medulla oblongata, and connected by twigs with the vagus. But take his two rules, at the close of the article, for the prevention of death during the extraction of teeth under chloroform, *i. e.* "wet the patient thoroughly over and lay him in a horizontal position."—Monthly Review of Dental Surgery, London. The writer speaks of cases of death having occurred in the extraction of teeth from chloroform, and thinks that the cause of death is from reflex action on the heart from not having taken enough; but he has not introduced any proof of this fact in the cases referred to. We all have heard of death from this drug where no surgical operation had been performed at all, hence these assumptions may or may not be true. I have no knowledge of a case of death from reflex action from the extraction of a tooth, when nothing had been given. Prof. Syms recommends the use of the drug with a free hand, in the extraction of teeth. Still I do not think the ends justify the means in lighter minor surgery of any kind. His two rules are objectionable: first in a dental office it is not convenient or practical to put the patient in a horizontal position for tooth extraction. Secondly, it would be dangerous from blood entering the wind pipe, and the tooth or fang escaping from the forceps and entering the wind pipe and causing death from asphyxia. In a hospital, patients might be put in the horizontal position when teeth are extracted.

The main question is, whether or not, we are justified in giving a dangerous drug to relieve pain in so simple an operation as that of extracting teeth, the shock of which can not be regarded as dangerous to the heart's action under any ordinary circumstances.

